# Reassessing pattern separation in the dentate gyrus

**DOI:** 10.3389/fnbeh.2013.00096

**Published:** 2013-07-30

**Authors:** Adam Santoro

**Affiliations:** Institute of Medical Science, University of TorontoToronto, ON, Canada

## Introduction

The dentate gyrus (DG) is postulated to be a “pattern separator” (Marr, 1971; Rolls, 1989a,b, 1990; Treves and Rolls, 1994). Yet, the definition of pattern separation has become a haze, with researchers using the term interchangeably to describe computational processes, changes in cell ensemble activity, and even behavioral phenomena (Leutgeb et al., 2007; McHugh et al., 2007; Clelland et al., 2009; Bakker et al., 2010). To accurately assess the large influx of papers purporting to attribute pattern separation to the DG, the concept must be reassessed; the original definition of pattern separation as a computational process and its newer, colloquial definition as a form of behavioral context discrimination must be accurately parsed if relations between the data are to be made.

## Pattern separation as a computational process

The work of Marr, Rolls, and Treves outlined the potential role of the DG as a pattern separator (Marr, 1971; Rolls, 1989a,b, 1990; Treves and Rolls, 1994). In trying to define a role for the hippocampus in memory formation, these researchers merged known facts about hippocampal physiology and anatomy with their insights of neural networks to produce a computational theory of learning in the hippocampus. It was noted that the representation of information is rarely, if ever, reduced to the level of the single neuron (Rolls, 1990). Instead, ensemble encoding is a much more valuable method of information storage. By encoding information at the cell-population level, properties such as pattern completion and generalization are possible. According to their theory, the CA3 region of the hippocampus acts as an auto-association matrix memory network. Information flows to the CA3 from the entorhinal cortex (EC) via the perforant path and from the DG via mossy fibers, and the CA3 encodes the input information as a unique firing pattern of pyramidal cells. The encoding CA3 cells are then able to establish Hebbian learning between each other by virtue of their dense recurrent collaterals. Thus, when the CA3 is presented with an incomplete set of input information (such as during a behavioral cue event), activation of a subset of pyramidal cells serves to elicit activity of the remaining CA3 cells that were involved in the initial encoding (pattern completion). The process of pattern completion illustrates a strength of ensemble encoding, and helps model the hippocampus involvement in a variety of cued memory tasks.

The computational models sought to establish an efficient encoding method. That is, they required that ensemble activity in the CA3 was sufficiently non-redundant given similar input information in order to reduce interference between separately encoded information. They postulated that the DG could function to orthogonalize inputs to the CA3 neural network. The DG is ideally suited for this role; a DG granule cell has a low contact probability with any given CA3 pyramidal cell, which means that two distinct subsets of granule cell activity will elicit unique cell-population activity in the CA3 (Rolls, 1990). Given the large number of granule cells relative to CA3 cells (Amaral et al., 2007), and the sparse nature of CA3 encoding, it is highly probable that any given inputs to the hippocampus, regardless of their similarity, will establish non-overlapping sets of encoding ensembles in the CA3. Thus, similar patterns of input information can be separated into distinct output ensembles.

## Pattern separation is realized at the cell population level

**Figure 1:**
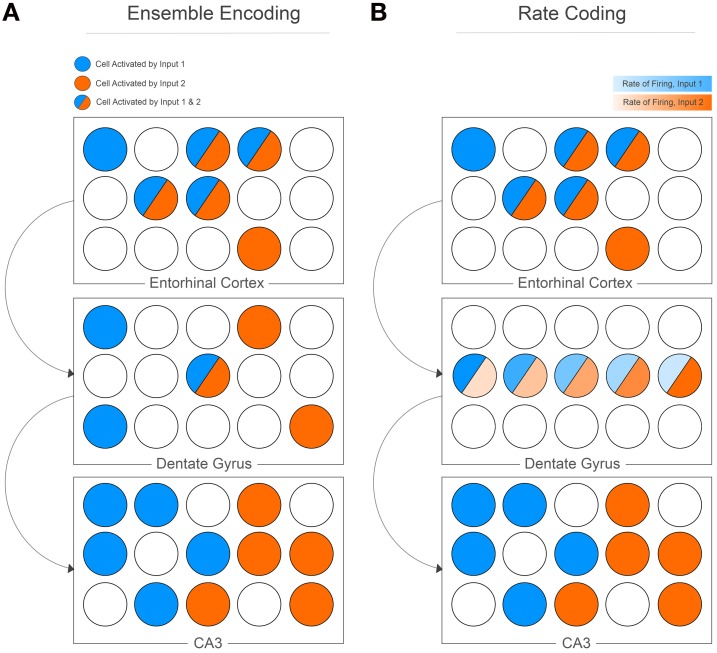
**Pattern separation was originally proposed as orthogonalization manifested at the cell population level (A)**. Cell population activity in the EC highly overlaps, but sparse activation of the DG and low contact probability of mossy fibers to CA3 cells manifests as unique cell ensemble activity for two separate input patterns. The DG can potentially encode information using rate coding **(B)**, whereby the same cells are active for separate inputs, but the rate of firing for each cell differs between inputs. The CA3 then decodes these rates to produce unique cell ensemble activity.

The computational work outlines pattern separation as it pertains to neural networks; input information is represented as either similar or dissimilar vectors, the CA3 memory network is an algebraic matrix, and the outputs of the matrix are orthogonalized vectors. Despite the mathematical underpinnings, the insights of the model are obvious, powerful, and clever. Most importantly for this opinion, however, is the fact that pattern separation is realized at the cell population level. Given an input from the EC, the ultimate goal is unique ensemble encoding at the level of the CA3 (Figure 1A). Consider input information from the EC, described as a subset of cell activity. The similarity of inputs from the EC can be defined using an arbitrary function (S) that measures the degree of overlap between the cell populations that are active given two input events (e.g., the Hamming distance), such that:

$$\text{Similarity}=S_{EC}\left(I_{1},\;I_{2}\right)$$

The computational models state that similar inputs from the EC will produce orthogonalized outputs in CA3. Thus, if *S*_*EC*_(*I*_1_, *I*_2_) is high (high similarity), then *S*_*CA*3_(*I*_1_, *I*_2_) should be low (low similarity). This implies that a function of the DG is to decrease the similarity of its input, such that:

$$S_{EC}\left(I_{1},\;I_{2}\right)\;>\;S_{DG}\left(I_{1},\;I_{2}\right)$$

And thus,

$$S_{EC}\left(I_{1},\;I_{2}\right)\;>\;S_{DG}\left(I_{1},\;I_{2}\right)\;>\;S_{CA3}\left(I_{1},\;I_{2}\right)$$

Although it may be inherent in the architecture of the DG that it may pattern separate via its mossy fiber connections [i.e., it is necessarily true that *S*_*DG*_(*I*_1_, *I*_2_) > *S*_*CA*3_(*I*_1_, *I*_2_)], it is imperative to note that there are conditions whereby this separation may be arbitrary, and even unnecessary. Consider a situation where *S*_*EC*_(*I*_1_, *I*_2_) = 0 (thus, each population is represented by a totally unique set of cells, or at the very least contain overlap simply arising due to chance). Any further separation accomplished by the DG is pointless, since *S*_*CA*3_(*I*_1_, *I*_2_) = 0 by virtue of the EC, and not the DG. There are a few important implications from this analysis. Firstly, pattern separation is not a phenomenon unique to only a few regions, such as the DG (Yassa and Stark, 2011). Rather, the *degree of pattern separation* (or its converse, *pattern convergence*) should be considered a measureable property of *any* region in a network. Secondly, pattern separation defined at the cell population level is inherently an *inter*-regional phenomenon, since input similarities must be measured against output similarities. Thus, to establish a region as a pattern separator, one must analyze the similarity of its input region as well as its output region. It is not sufficient to simply measure the similarity of the output, since one can imagine a situation where a region necessarily receives already separated information. Considering the very nature of our sensory system (different sensory stimuli elicit different stimuli sensing cells) it is logical to assume that pattern separation can occur serially before reaching the DG, in the sensing organs, relay nuclei, sensory cortices, etc. In fact, sparse coding for visual processing has been illustrated in the primary visual cortex in macaques (Vinje and Gallant, 2000).

The third implication has some important corollaries when thought of in the context of pattern completion. Pattern completion is often thought of as an opposite of pattern separation. However, a true converse to pattern separation is pattern convergence, which is a distinctly unique process from pattern completion. In the case of pattern convergence, as information flows from region A to region B:

$$S_{A}\left(I_{1},\;I_{2}\right)\;<\;S_{B}\left(I_{1},\;I_{2}\right)$$

Thus, cell population activity becomes *more* similar when progressing from region A to region B, representing a convergence of patterns. The concept of pattern separation and convergence can be defined for any region; in fact, every region is necessarily a pattern separator or converger to some degree. Pattern completion is necessarily an *intra*-regional phenomenon. Pattern completion defines the state of a network, given a certain input, as it changes over time. Formally stated, pattern completion states that:

$$S_{A}\left(I_{1^{\prime }},\;I_{1}\right)\;↑\;as\;t\to \infty$$

In other words, the cell population evoked by a subset of a given input (*I*_1′_) shifts toward the cell population evoked by the originally encoded input (*I*_1_) as time elapses.

To date, no study has *directly* confirmed the existence of a pattern separation mechanism at the cell population level in the EC-DG-CA3 circuit—although, there is some indirect evidence. In an interesting study Bakker *et al*. used fMRI to analyze activity in the DG/CA3 during an incidental memory encoding task (Bakker et al., 2010). Participants were presented with a series of images that were either novel, repetitions of previously seen images, or slight variations of previously seen images (lures). They hypothesized that “… if a given subregion was engaged in processes of pattern separation, the lure would more likely be treated like a new stimulus than a repetition and show activity similar to that for a first presentation of astimulus.” Indeed, this is what they found; lure images resulted in activity in the DG/CA3 region that equalled activity evoked by novel images, and both lure and novel images evoked greater activity as compared to repetitions. More importantly, activity in the EC evoked by lures was similar to that evoked by repetitions. Since it can be (generally) assumed that novel stimuli induce unique *cellular* activity, and that repeated stimuli induce suppressed *fMRI* activity relative to novel stimuli, it can be inferred that the DG/CA3 region potentially utilizes unique cellular representations for similar input stimuli, and that the EC uses similar cellular representations for similar stimuli. This interpretation is consistent with a pattern separation mechanism at the cellular level. However, since cell population activity was not directly recorded in each of the EC, DG, and CA3, this evidence is an indirect, and it can still not be conclusively stated that a pattern separation mechanism occurs in the DG.

## Pattern separation not at the cell population level

It is easy to imagine a situation whereby pattern separation is not described at the cell population level. Originally evoked as a derivation of orthogonalization, pattern separation can represent any equivalent process whereby computational orthogonalization is realized. For example, consider a learning matrix that models inputs to a single cell, with the output vector describing the resulting firing rate of the cell. Given certain inputs to its dendrites, a pattern separator can function to adequately separate resulting firing rates given similar inputs. An elegant study by Leutgeb and colleagues found that a similar population of cells in the DG of rats is recruited when they are placed in similar environments (a gradually morphing box); however, the rate of firing of these cells changes in each environment (Leutgeb et al., 2007). They conclude that “The divergent direction of the rate changes in the different firing fields of dentate cells accentuates the decorrelation of the ensemble activity, which allows each environment to be represented by a unique rate pattern in a small number of granule cells.” The CA3 is then able to disambiguate this rate mapping, resulting in unique ensemble activity in the CA3 in similar environments (Figure 1B). This is not the original realization of pattern separation that the computational models had in mind, since it addresses rate coding in the DG rather than ensemble encoding. Thus, although the data are powerful, care needs to be taken when relating the results to the original computational models; they both address a role for the DG in pattern separation, but tackle the issue from different directions. The models do not validate the data, and the data do not validate the models, suggesting that the original computation models may need to be reformulated to consider a more comprehensive account of the nature encoding in the hippocampus.

## Pattern separation as a behavioral phenomenon

A recent trend in the pattern separation literature is to define pattern separation as the literal behavioral ability to discriminate related stimuli (often also referred to as “behavioral pattern separation,” or “spatial pattern separation,” and even simply “pattern separation”) (Clelland et al., 2009). For example, consider a task in which an animal needs to detect environmental similarities and respond appropriately (such as during a touch screen task, or during contextual fear conditioning discrimination). The animal needs to cognitively separate similar input patterns so that it can behave differently these similar patterns. Pattern separation at the level of the cell ensemble is thought to be a potential mechanism for behavioral pattern separation, but the two phenomena are fundamentally different—the former is defined in terms of cell ensembles, and the latter in terms of behavior. As such behavioral pattern separation is *consistent* with pattern separation of cell ensembles, but it does not *entail* it. This is an important distinction, as it implies that behavioral pattern separation tasks cannot be used as correlates of, or cannot be used to infer the existence of cell ensemble pattern separation until direct evidence for the causal relationship between cell ensemble pattern separation and behavioral pattern separation is established. To properly conclude that behavioral discrimination entails pattern separation at the level of the cell ensemble in the DG, the following should occur: (1) population activity in the EC, DG, and CA3 is measured during performance of a discrimination task, and (2) causality is determined by manipulating cellular pattern separation. As such, there is a need for care when making conclusions from behavioral data, and when relating behavioral data to electrophysiological data and computation models.

## Operational definitions for pattern separation

Pattern separation can be defined computationally, at the level of the cell population, at the level of the single cell, or behaviorally. Given the complex nature of the term and the connotations that it carries, it is important to establish some operational definitions. Here are some suggestions:

1. The term “pattern separation” should refer to the manifestation of computational orthogonalization, whereby overlapping cell populations corresponding to given input stimuli diverge as population activity is measured across regions. As stated previously, this formalizes as:
  $$S_{A}\left(I_{1},\;I_{2}\right)\;>\;S_{B}\left(I_{1},\;I_{2}\right)\;>\;S_{C}\left(I_{1},\;I_{2}\right)$$
2. The term “behavioral pattern separation” should no longer be used, as it is synonymous with “behavioral discrimination.” Both terms are consistent with a pattern separation mechanism at the level of the cell ensemble, but the term “behavioral pattern separation” is often mistakenly interpreted as synonymous with the definition stated in #1, and even as synonymous with orthogonalization. Thus, the term “behavioral discrimination,” or just “discrimination,” should be used when describing this behavioral phenomenon.

Although computational modeling of ensemble activity, rate coding of neurons, and behavioral discrimination are each potentially linked to pattern separation, they are not equivalent processes, and thus cannot be used interchangeably under the umbrella term “pattern separation.”
